# Improving Data Quality in Clinical Research Informatics Tools

**DOI:** 10.3389/fdata.2022.871897

**Published:** 2022-04-29

**Authors:** Ahmed AbuHalimeh

**Affiliations:** Information Science Department, University of Arkansas at Little Rock, Little Rock, AR, United States

**Keywords:** clinical research data, data quality, research informatics, informatics, management of clinical data

## Abstract

Maintaining data quality is a fundamental requirement for any successful and long-term data management. Providing high-quality, reliable, and statistically sound data is a primary goal for clinical research informatics. In addition, effective data governance and management are essential to ensuring accurate data counts, reports, and validation. As a crucial step of the clinical research process, it is important to establish and maintain organization-wide standards for data quality management to ensure consistency across all systems designed primarily for cohort identification, allowing users to perform an enterprise-wide search on a clinical research data repository to determine the existence of a set of patients meeting certain inclusion or exclusion criteria. Some of the clinical research tools are referred to as de-identified data tools. Assessing and improving the quality of data used by clinical research informatics tools are both important and difficult tasks. For an increasing number of users who rely on information as one of their most important assets, enforcing high data quality levels represents a strategic investment to preserve the value of the data. In clinical research informatics, better data quality translates into better research results and better patient care. However, achieving high-quality data standards is a major task because of the variety of ways that errors might be introduced in a system and the difficulty of correcting them systematically. Problems with data quality tend to fall into two categories. The first category is related to inconsistency among data resources such as format, syntax, and semantic inconsistencies. The second category is related to poor ETL and data mapping processes. In this paper, we describe a real-life case study on assessing and improving the data quality at one of healthcare organizations. This paper compares between the results obtained from two de-identified data systems i2b2, and Epic Slicedicer, and discuss the data quality dimensions' specific to the clinical research informatics context, and the possible data quality issues between the de-identified systems. This work in paper aims to propose steps/rules for maintaining the data quality among different systems to help data managers, information systems teams, and informaticists at any health care organization to monitor and sustain data quality as part of their business intelligence, data governance, and data democratization processes.

## Introduction

Data is the building block in all research, as results are only as good as the data upon which the conclusions were formed. However, researchers may receive minimal training on how to use the de-identified data systems and methods for achieving, assessing, or controlling the quality of research data (Nahm, 2012; Zozus et al., 2019).

De-identified data systems are defined as systems/tools that allow users to drag and drop search terms from a hierarchical ontology into a Venn diagram-like interface. Investigators can perform an initial analysis on the de-identified cohort. Furthermore, de-identified data systems have no features to indicate the data quality or assist in identifying the data quality; these systems only provide counts.

Informatics is the science of how to use data, information, and knowledge to improve human health and the delivery of healthcare services (American Medical Informatics Association, 2022).

Clinical Informatics is the application of informatics and information technology to deliver healthcare services. For example, patient portals, electronic medical records (EMRs), telehealth, healthcare apps, and a variety of data reporting tools (American Medical Informatics Association, 2022).

The case presented in this paper focuses on the quality of data obtained from two de-identified systems (Epic Slicerdicer and i2b2).The purpose of this paper is to discuss the quality of the data (counts) generated from the two systems, understand the potential causes of the data quality issues, and propose steps to improve the quality and increase the trust of the generated counts by comparing the accuracy, consistency, validity, and understandability of the outcomes from the two systems.

The proposed steps for maintaining the data quality among different systems aim to help data managers, information systems teams, and informaticists at a healthcare organization monitor and sustain data quality as part of their business intelligence, data governance, and data democratization processes. The quality improvement steps proposed are generic and contributes in adding generic and essential steps to automate data curation and data governance to tackle various data quality problem.

The remainder of this paper is organized as follows. In the following section, we introduce the importance of data quality to clinical research informatics, the study case and study method and materials presented in the Importance of Data Quality to Clinical Research Informatics, Case Study Goals, and Methodology section. The findings and the discussion part, and the proposed steps to ensure data quality are discussed in Discussion section. Conclusions are drawn and work contribution is discussed in Conclusion section.

## Importance of Data Quality to Clinical Research Informatics

Data quality refers to the degree data meets the expectations of data consumers and their intended use of the data (Pipino et al., 2002; Halimeh, 2011; AbuHalimeh and Tudoreanu, 2014). In clinical informatics, this depends on the study conducted (Nahm, 2012; Zozus et al., 2019).

**Figure 1:**
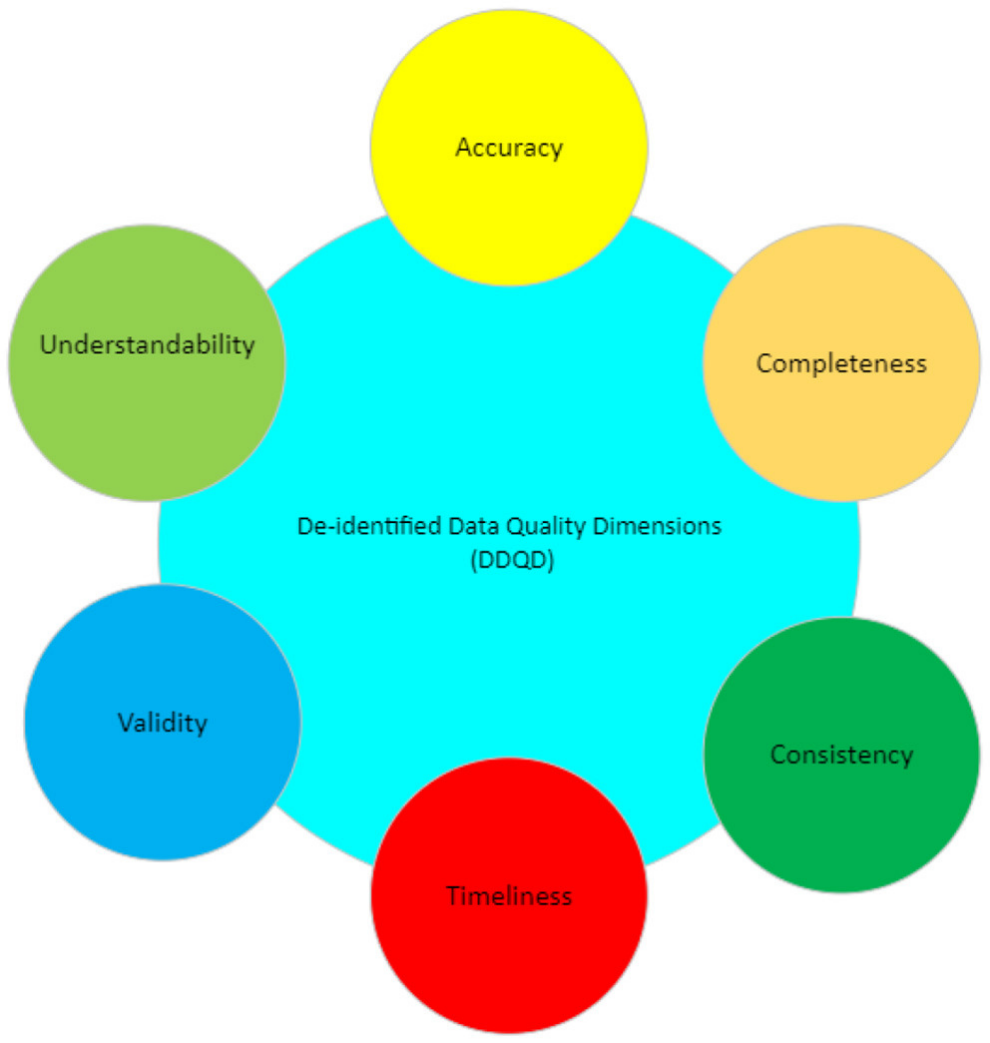
De-identified data quality dimensions (DDQD).

The meaning of data quality lies in how the data is perceived and used by its consumer. Identifying data quality involves two stages: first, highlighting which characteristics (Dimensions) are important (Figure 1) and second, determining how these dimensions affect the population in question (Halimeh, 2011; AbuHalimeh and Tudoreanu, 2014).

This paper focuses on a subset of data quality dimensions, which we term de-identified data quality dimensions (DDQD). We think these dimensions should be mainly considered to maintain the data quality in de-identified systems because the absence of any of these dimensions will affect the overall quality of the data in the de-identified data systems. These dimensions are described in Table 1 below.

**Table 1:** De-identified data quality dimensions definitions (DDQD).

**Quality dimension**	**Definition**
Accuracy	Refers to the degree to which information accurately reflects an event or object described How well does a piece of information reflect reality?
Completeness	Refers to the extent to which data is not missing and of sufficient amount for the task at hand Does it fulfill data consumer's expectations? The needed amount is known?
Consistency	Refers to the extent the is applicable and helpful to the task at hand Does information stored in one place match relevant data stored elsewhere?
Timeliness	Refers to the extent to which the data is sufficiently up- to-date for the task at hand Is the data available up-to-date when you need it?
Validity	Refers to information that doesn't conform to a specific format or doesn't follow business rules Is information in a specific format, does it follow business rules, or is it in an unusable format?
Understandability	Refers to the degree the data can be comprehended Can the user understand the data easily?

The impact of quality data and management is in performance and efficiency gains and the ability to extract new understandings. Poor clinical informatics data quality can cause glitches throughout an organization. This impact includes the quality of research outcomes, healthcare services, and decision-making.

Quality is not a simple scalar measure but can be defined on multiple dimensions, with each dimension yielding different meanings to different information consumers and processes (Halimeh, 2011; AbuHalimeh and Tudoreanu, 2014). Each dimension can be measured and assessed differently. Data quality assessment implies providing a value for each dimension about how much of the dimension or quality feature is achieved to enable adequate understanding and management. Data quality and the discipline of informatics are undistinguishable interconnected. Data quality depends on how data are collected, processed, and presented; this is what makes data quality very important and sometimes complicated because data collection and processing varies from one study to another. Clinical informatics data can include different data formats and types and could come from different resources.

## Case Study Goals

The primary goal is to compare, identify and understand discrepancies in a patient count in i2b2 compared to Epic Slicerdicer (Galaxy, 2021). The secondary goal was to create a data dictionary that clinical researchers would easily understand. For example, if they wanted a count of patients with asthma, they would know (1) what diagnoses were used to identify patients, (2) where these diagnoses were captured, and (3) that this count matched existing clinical knowledge.

The case described below is from one of the healthcare organizations wanted to have the ability to ingest other sources of research-specific data, such as genomic information, and the existing products did not have a way to do that. After deliberation i2b2 (The i2b2 tranSMART Foundation, 2021) was chosen as the data model for their clinical data warehouse. Prior to going live with users, however, it is very important and essential to validate that the data in their Clinical Data Warehouse (CDW) was accurate.

## Methodology

### Participants

The clinical validation process involved a clinical informatician, data analyst, and ETL developer.

### Data

Many healthcare organizations use at least one of the three Epic databases (Chronicles, Clarity, and Caboodle). The data source used to feed i2b2 and Slicerdicer tools was Caboodle database.

### Tools

The tools used to perform the study are i2b2 tool and Epic Slicerdicer.

I2b2: Informatics for Integrating Biology and the Bedside (i2b2) is an open-source clinical data warehousing and analytics research platform; i2b2 enables sharing, integration, standardization, and analysis of heterogeneous data from healthcare and research (The i2b2 tranSMART Foundation, 2021).

Epic Slicerdicer: is a self-service reporting tool that allows physicians ready access to clinical data that is customizable by patient populations for data exploration. Slicerdicer allows the user to choose and search a specific patient population to answer questions about diagnoses, demographics, and procedures performed (Galaxy, 2021).

### Method Description

The study was designed in a way to compare, identify and understand discrepancies in a patient count in i2b2 compared to Epic Slicerdicer (Galaxy, 2021). We achieved this goal by choosing a task based on the nature of the tools.

The first step was by running the same query to look at patient demographics (race, ethnicity, gender) and identified different aggregations with race and ethnicity in i2b2 compared with Slicerdicer, which was more granular as shown in Table 2. For example, Cuban and Puerto Rican values in Slicerdicer were included in the Other Hispanic or Latino category in i2b2. The discrepancies are shown in Table 2.

**Table 2:** Patients demographic counts.

**Patient counts**	**i2b2**	**Epic Slicerdicer**	**% different**
**Race**			
American Indian or Alaska Native	1,434	1,579	9%
Asian	7,051	7,480	6%
Asian Indian		917	
Chinese		177	
Filipino		148	
Japanese		30	
Korean		62	
Other Asian		6,146	
Black or African American	2,38,638	2,42,871	2%
Native Hawaiian or Other Pacific Islander	2,990	3,430	13%
Native Hawaiian		170	
Guamanian or Chamorro		21	
Samoan		14	
Other Pacific Islander		2,582	
Multiple race		^*^	
Other	99,081	1,07,759	8%
Unknown		31,733	
Decline to answer		176	
White	6,59,140	6,70,182	2%
**Ethnicity**			
Hispanic or Latino	61,237	64,354	5%
Other Hispanic or Latino		21,021	
Mexican, Mexican American, or Chicano/a		2,263	
Puerto Rican		118	
Cuban		41	
Non-Hispanic or Latino	2,63,119	2,81,091	6%
Unknown	7,33,886	7,69,097	5%
None of the above		7,30,480	
Decline to answer		300	
**Gender**			
Female	5,36,450	5,45,895	2%
Male	5,55,851	5,68,026	2%
Unknown	548	615	11%
Other		5	

* *Note, if patients have more than one entry for race data, Slicerdicer counts them in all of the selected fields*.

The second steps was running same query to explore diagnoses using J45^*^ as the ICD-10 code for asthma and Type 1 diabetes diagnosis code (E10^*^) as shown in Table 3.

**Table 3:** Patients count based on diagnosis codes.

**Patient counts**	**i2b2**	**Epic Slicerdicer**	**% different**
Asthma (J45^*^)			
Diagnosis	14,500	23,958	39.48%
Billing diagnosis	20,429	22,265	8.25%
Type 1 diabetes (E10^*^)			
Diagnosis	1,900	2,202	13.71%
Billing diagnosis	1,869	2,025	7.70%

* *Indicates multiple race, the category exist in Epic slicer Dicer is not available in i2b2*.

The Percentage Difference Calculator (% difference calculator) was implemented to find the percent difference between i2b2 counts and Epic Slicerdicer counts >0. The percentage difference as described in the formula below is usually calculated when you want to know the difference in percentage between two numbers is used to estimate the quality of the counts coming from the two tools, the threshold for accepted quality in this study was below 2% difference.

*V*_1_ = i2b2 counts and *V*_2_ = Slicerdicer counts and counts are plugged into the below formula


$$\begin{array}{l} Percentage\;difference\;=\;|V1-V2|/\left[\left(V1\;+\;V2\right)/2\right]\;\times \;100 \end{array}$$

A paired *t*-test is used to investigate the difference between two counts from i2b2 and Epic Slicerdicer for the same query.

### Findings

All the results obtained from comparing the counts between Slicerdicer and i2b2 are listed in the Tables 2, 3 below.

However, when diagnoses were explored, larger discrepancies were noted. There are 2 diagnosis fields in i2b2, one for billing diagnosis, and one for diagnosis. Using J45^*^ as the ICD-10 code for asthma resulted in 22,265 patients when using the billing diagnosis code in Slicerdicer but only 20,429 in i2b2. The discrepancy using diagnosis was even larger. Patient count results for Type 1 diabetes diagnosis code (E10^*^) using both diagnosis and billing are also shown in Table 3.

The best approach to understand the reasons of this discrepancy was by looking at the diagnosis options in Slicerdicer to build a hypothesis on where this discrepancy might come from. Next, was examining the SQL code for the Caboodle to i2b2 ETL process.

### Hypotheses

The following hypotheses were considered:

H0: There is no discrepancy in the data elements used to pull the data.

H1: There is a discrepancy in the data elements used to pull the data.

Paired sample *t*-test was implemented on the counts obtained from the ib2b and Slicerdicer using different data points. The *p*-value was equals to 0, [*P*(x ≤ –Infinity) = 0] in all cases that means that the chance of type I error (rejecting a correct H0) is small: 0 (0%). The smaller the *p*-value the more it supports H1. For example results of the paired *t*-test indicated that there is a significant medium difference between i2b2 (*M* = 14,500, *SD* = 0) and Epic Slicerdicer (*M* = 23,958, *SD* = 0), t(0) = Infinity, *p* < 0.001 and results of the paired *t*-test indicated that there is a significant medium difference between i2b2 (*M* = 1,55,434, *SD* = 0) and Epic Slicerdicer (*M* = 1,579, *SD* = 0), t(0) = Infinity, *p* < 0.001.

Since the *p*-value < α, H0 is rejected the i2b2 population's average is considered to be not equal to the Epic Slicerdicer population's average. In other words, the difference between the averages of i2b2 and Epic Slicerdicer is big enough to be statistically significant.

The paired *t*-test results supported the alternative hypothesis and revealed that there is a discrepancy in the data elements used to pull the data.

Also the Percentage Difference Calculator (% difference calculator) results which used to estimate the quality of the counts coming from the two tools, the majority of the results exceeded the threshold for accepted quality in this study (below 2%) difference as shown in Tables 2, 3. The percentage difference results showed and provided a strong evidence for a crucial quality issue in the counts obtained.

In that process of examining the SQL code for the Caboodle to i2b2 ETL process, the SQL code results showed the code only looked at billing and encounter diagnosis and everything that was not a billing diagnosis was labeled diagnosis. Slicerdicer and even Caboodle include other diagnosis sources such as medical history, hospital problem, and problem list. This was included in the data dictionary so that researchers would understand what sources i2b2 was using and that if they wanted data beyond that, they would have to request data from Caboodle.

## Discussion

The discrepancies led to major information quality issues such as data inconsistency and data accuracy both affects the believability and the validity of the data which also are major data quality measures. The discrepancies noted above are likely due to several factors. First, Slicerdicer counts patients for every race selected instead of i2b2, which only takes the first race field, this because two data models were used to pattern race and ethnicity variables in i2b2 to the 1997 OMB race categories and the 2003 OMB variables, which contains a more granular set of race and ethnicity categories. The mapping then was done to ‘bundle” the other races to a more general set of categories. This could be the reason why there is a reduction of concepts because maybe the map is incomplete.

Secondly, the purpose of the Extract-Load-Transform (ETL) process is to load the warehouse with integrated and cleansed data. Data quality focuses on the contents of the individual records to ensure the data loaded into the target destination is accurate, reliable, and consistent, so the ETL code should be evaluated to ensure the data extracted generally match what researchers want. In our case, understanding what diagnosis most researchers are interested in—they may want encounter diagnosis instead of including problem list and medical history. Thirdly, the causes for data quality issues are format differences or conversion errors (Azeroual et al., 2019; Souibgui et al., 2019).

Lastly, data loss could be present in the ETL process, which is one of the challenges in ETL processes because of the nature of the source systems. Data losses arise from the disparities among the source operational systems. Source systems are very diverse and disparate because of the increased amount of data, modification of data formats, and modification of and deriving new data elements.

In general, data integration with heterogeneous systems is not an easy task. This is mainly due to the fact that many data exchange channels must be developed in order to allow an exchange of data between the systems (Berkhoff et al., 2012) and to solve problems related to the provision of interoperability between systems on the level of data (Macura, 2014).

### Steps to Ensure Informatics Quality

To improve the data quality generated from the de-identified systems which is mainly counts, and to solve any data quality issues related to the provision of interoperability between the used tools on the level of data, we propose the following steps:

1. Make data “fit for use.”
  To make data fit for use, data governance bodies must clearly define major data concepts/variables included in the de-identified systems and standardize their collection and monitoring processes; this can increase clinical data reliability and reduce the inconsistency of data quality among systems involved (Halimeh, 2011; AbuHalimeh and Tudoreanu, 2014).
2. Define data elements (data dictionary).
  This is a fundamental part—the lack of clear definitions of source data and controlled data collection procedures often raises concerns about the quality of data provided in such environments and, consequently, about the evidence level of related findings (Spengler et al., 2020). Developing a data dictionary is essential to ensuring data quality, especially in de-identified systems where all data elements are aggregated in a specific way, and there are not enough details about each concept. A data dictionary will serve as a guidebook to define the major data concepts. To do this, organizations must determine what data about data (metadata) is helpful to the researchers when they use the de-identified data systems. In addition, identifying more targeted data concepts and process workflows can help reduce some of the time and effort for researchers when working with large amounts of data and ultimately improve overall data quality.
3. Applying good ETL practices such as data cleansing mechanisms to get the data to a place that acts well with data from other sources.
4. Choose smart ETL architecture that allows you to update components of your ETL process when data and systems need change or update to prevent any data loss and to ensure data integrity and consistency.
5. Apply Data Lineage techniques. This will help in understanding where data originated from, when it was loaded, how it was transformed and is essential for the integrity of the downstream data and the process that moves it to any of the de-identified system.
6. Establish a process for cleansing and tracing suspicious data and unusual rows of data when are revealed.
7. Users need to revise their queries and refine results as they combine data variables.
8. Having a clinical informaticist on board can also be beneficial to the process. They can ensure that your data reflects what is seen in clinical practice or help explain questionable data with their knowledge of clinical workflows and how that data is collected, especially if your analyst has no clinical background.

## Conclusion

The success of any de-identified data tool depends largely on the quality of the data used, and the mapping process which is intertwined with the extraction and transformation components. The ETL process is a crucial component in determining the quality of the data generated by an information system.

This study proved that the discrepancies in the data used in the data pull process led to major information quality issues such as data inconsistency and data accuracy which both affects the believability and the validity of the data which also are major data quality measures.

Our contribution in this paper is to propose a set of steps that together form guidelines for a method or automated procedures and tools to manage data quality and data governance in a multifaceted, diverse information environment such as healthcare organizations and to enhance the data quality among the de-identified data tools.

Future plan is to study more clinical informatics tools such TriNetX and other sets of medical data to assess the quality of the counts obtained from these tools.

## Author Contributions

The author confirms being the sole contributor of this work and has approved it for publication.

## Conflict of Interest

The author declares that the research was conducted in the absence of any commercial or financial relationships that could be construed as a potential conflict of interest.

## Publisher's Note

All claims expressed in this article are solely those of the authors and do not necessarily represent those of their affiliated organizations, or those of the publisher, the editors and the reviewers. Any product that may be evaluated in this article, or claim that may be made by its manufacturer, is not guaranteed or endorsed by the publisher.
